# Extracellular Domain of IL-10 Receptor Chain-2 (IL-10R2) and Its Arginine-Containing Peptides Are Susceptible Substrates for Human Prostate Kallikrein-2 (KLK2)

**DOI:** 10.1021/acs.biochem.4c00292

**Published:** 2024-08-06

**Authors:** Juliana R. Oliveira, José Thalles Lacerda, Tarciso A. Sellani, Elaine G. Rodrigues, Luiz R. Travassos, Maria A. Juliano, Luiz Juliano

**Affiliations:** † Department of Biophysics, Escola Paulista de Medicina, Federal University of São Paulo, Rua Três de Maio 100, São Paulo 04044-20, Brazil; ‡ International Research Center, A.C. Camargo Cancer Center, Rua Taguá, 440, São Paulo 01509-010, Brazil; § Department of Microbiology, Immunology and Parasitology, Escola Paulista de Medicina, Federal University of São Paulo, Rua Botucatu 862, São Paulo 04023-901, Brazil

## Abstract

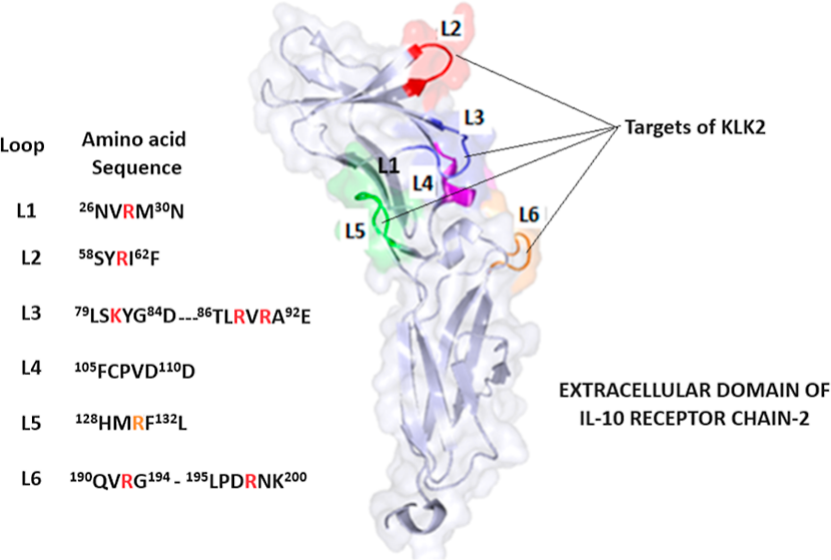

The kallikrein-related peptidase KLK2 has restricted expression in the prostate luminal epithelium, and its protein target is unknown. The present work reports the hydrolytic activities of KLK2 on libraries of fluorescence resonance energy-transfer peptides from which the sequence SYRIF was the most susceptible substrate for KLK2. The sequence SYRIF is present at the extracellular *N*-terminal segment (^58^SYRIF^63^Q) of IL-10R2. KLK2 was fully active at pH 8.0–8.2, found only in prostate inflammatory conditions, and strongly activated by sodium citrate and glycosaminoglycans, the quantities and structures controlled by prostate cells. Bone-marrow-derived macrophages (BMDM) have IL-10R2 expressed on the cell surface, which is significantly reduced after KLK2 treatment, as determined by flow cytometry (FACS analysis). The IL-10 inhibition of the inflammatory response to LPS/IFN-γ in BMDM cells due to decreased nitric oxide, TNF-α, and IL-12 p40 levels is significantly reduced upon treatment of these cells with KLK2. Similar experiments with KLK3 did not show these effects. These observations indicate that KLK2 proteolytic activity plays a role in prostate inflammation and makes KLK2 a promising target for prostatitis treatment.

## Introduction

Human kallikrein-related peptidases KLK2 and KLK3 (PSA—prostatic-specific antigen) are closely related to prostate-restricted serine proteases, abundant in the luminal epithelium and secreted into the prostatic fluid. KLK2 is a trypsin-like serine protease that cleaves only after the basic amino acids arginine (R) or lysine (K).^1,2^ This KLK2 hydrolytic activity contrasts with the chymotrypsin-like KLK3 that instead cleaves bonds of hydrophobic amino acids.^3^ The functional proteases such as human kallikreins (KLKs) have a finely tuned substrate specificity that goes beyond the recognition of the amino acid at the cleavage site, extending it to the other amino acids on both sides of the hydrolysis site. In this regard, protease/substrate interaction requirements have to be considered as proposed by Schechter and Berger^4^ (1967), where S_*n*_···–S_3_–S_2_–S_1_–S_1_’–S_2_’–S_3_’–···S_*n*_’ are the substrate binding sites within the active site of the protease, and P_*n*_···–P_3_–P_2_–P_1_^↓^P_1_’–P_2_’–P_3_’–···P_*n*_’ are the substrate amino acid residues that bind to S_*n*_···–S_*n*_’ sites, the arrow indicating the cleavage site. Fluorescence resonance energy transfer (FRET) peptides are convenient substrates to map the specificity of nonprime (S) and prime (S′) binding sites of the protease active site, and Abz-peptidyl-Q-EDDnp FRET peptide concept is convenient as endoprotease substrates.^5^

In the present work, we explored the S_3_ to S_2_′ subsite preferences of KLK2 using five series of FRET peptides derived from the peptide Abz-KLRSSK-Q-EDDnp, cleaved at the R–S peptide bond by KLK2. We choose the sequence KLRSSK based on previously reported substrate subsite requirements of human KLK1,^6,7^ the reference member in the human KLK family. We synthesized and assayed the series: Abz-XLRSSK-Q-EDDnp (for S_3_), Abz-KXRSSK-Q-EDDnp (for S_2_), Abz-KLXSSK-Q-EDDnp (for S_1_), Abz-KLRXSK-Q-EDDnp (for S′_1_), and Abz-KLRSXK-Q-EDDnp (for S′_2_), where X represents a natural amino acid of each class, except cysteine. The amino acid sequence SYRIF corresponds to the amino acid in each series, resulting in higher hydrolysis. Using the basic local alignment search tool (BLAST), SYRIF appears in the extracellular domain of IL-10 receptor chain-2 (^58^SYRIF^63^Q), which could be proposed as a natural substrate for KLK2. Then, we synthesized the FRET peptide sequence (Abz- SYRIFQ-Q-EDDnp), resulting in the highly susceptible substrate for this protease. We have previously used a similar strategy with KLK6^8^ and KLK7,^9^ indicating the extracellular amino-terminal domain of human ionotropic glutamate receptor subunits and semaphorin 6B, respectively, as their natural substrates.

The modulation of KLK2 hydrolytic activity by sodium citrate, glycosaminoglycans (GAGs), and pH was also explored because the prostate has a peculiar extracellular environment as follows: (a) It is the only organ that concentrates large amounts of sodium citrate, a typical kosmotropic salt;^10^ (b) it has large amounts of different proteoglycans in epithelial cells and stroma;^11^ and (c) the pH of the healthy prostatic fluid is 6.5–6.7, but in inflammatory conditions, it reaches 8.1.^12,13^ Finally, using bone marrow-derived macrophages (BMDMs) that express IL-10 receptors on the cell surface, we evaluated its inactivation and concentration after KLK2 treatment.

## Materials and Methods

### Recombinant KLK2

Obtained from a baculovirus-insect cell line system, purified, and activated as previously reported,^14^ MUGB (4-methylumbelliferyl *p*-guanidino benzoate hydrochloride) was used for the spectrofluorimetric determination of molar concentrations of active KLK2.^15^ KLK3 was obtained and assayed as previously reported.^3^

### FRET Peptide Synthesis and Enzymatic Kinetic Measurements

All of the FRET peptides were obtained by solid-phase peptide synthesis. The purity of FRET peptides to 95% or higher was obtained by semipreparative HPLC on a C18 reversed-phase column and used as protease substrates, as previously reported.^5^ Details about the purification and quality of the FRET peptides are provided in the Supporting Information. The enzymatic kinetic measurements were all performed with FRET peptides as previously described.^5^

### Glycosaminoglycans

Bovine lung heparin was prepared as previously reported;^16^ dermatan sulfate (12 000 Da) and chondroitin sulfate (25 000 Da) were purchased from Seikagaku Kogyo Co. (Tokyo, Japan), and heparan sulfate (16 000 Da) from the bovine lung was a generous gift from Dr. P. Bianchini (Opocrin Research Laboratories, Modena, Italy).

### Macrophage Differentiation from Murine Bone Marrow Progenitors

BMDM were obtained from C57Bl/6 mice and used as previously reported.^17^

### Analysis of IL-10R2 Expression in BMDM

Cell surface markers were used to infer the expression of IL-10R2 in BMDM cells. We incubated these cells with KLK2 (7.5 μg/mL) or KLK3 (7.5 μg/mL) for 2 h. Cells were washed twice with PBS and 1% BSA-containing PBS and incubated for 1 h at 4 °C with an in-house-prepared normal C57Bl/6 mouse serum (1:30 diluted in PBS containing 1% BSA) to block Fc receptors. Cells were incubated for 1 h at 4 °C with APC-conjugated CD11b, PercP-Cy5-conjugated F4/80 surface antibodies (B.D. Bioscience), and AlexaFluor488-conjugated *anti*-IL-10RB/IL-10R2 (Abcam) for IL-10R2 expression in the BMDM evaluation. We acquired the data with a FACSCanto II instrument (B.D. Biosciences). For flow cytometric analyses, we used FlowJo Software (Tree Star, CA, USA), and fluorescence data was extracted as the geometric mean.^18^

### In Vitro Stimulation of BMDM

For BMDM activation assays in vitro, cells were plated at a density of 10^5^ cells/mL in 96-well plates at 37 °C in the absence and presence of KLK2 or KLK3 (7.5 μg/mL) for 2 h. The cells were then washed with PBS and treated with LPS (200 ng/mL)/IFN-γ (100 U/mL) stimuli in the presence or absence of recombinant IL-10 (10 ng/mL). The activation response of BMDM cells was evaluated after 72 h by quantification of nitric oxide (NO), TNF-α, and IL-12 p40 in the supernatant of BMDM cell cultures. NO was quantified using the Griess assay,^19^ and the cytokines TNF-α and IL-12 p40 were quantified using sandwich enzyme-linked immunosorbent assay (ELISA) kits (B.D. Biosciences) following the manufacturer’s instructions.

### Statistical Analysis

Statistical analyses were performed using an unpaired Student’s *t*-test for 2 groups comparison. For comparisons of 3 groups or more, we used the one-way ANOVA test, followed by Tukey’s multiple comparisons, as described in the figure legends. In all studies, a *p*-value <0.05 was considered statistically significant.

## Results

### Substrate Specificity of KLK2

Five series of FRET peptides derived from Abz-KLRSSK-Q-EDDnp were assayed with KLK2 in 20 mM Tris–HCl, pH 8.0, and 1 M sodium citrate. The specificity of the S_1_ subsite was explored with the peptide series Abz-KLXSSK-Q-EDDnp, where X = amino acid R, A, N, D, F, G, Q, E, H, I, L, M, P, S, Y, T, V, or K. Only the peptide Abz-KLRSSKQ-EDDnp was hydrolyzed (*k*_cat_ = 11.3 s^–1^, *K*_m_ = 6.5 μM, and *k*_cat_/*K*_m_ = 1738 mM^–1^ s^–1^). The specificity of subsites S_3_, S_2_, S_1_’, and S_2_’ was explored by the FRET peptide series Abz-XLRSSKQ-EDDnp (for S_3_), Abz-KXRSSKQ-EDDnp (for S_2_), Abz-KLRXSKQ-EDDnp (for S_1_’), and Abz-KLRSXKQ-EDDnp (for S_2_’). All of them were hydrolyzed only at R–S or R–X peptide bonds, and the kinetic parameters for their hydrolysis by KLK2 are listed in Table 1.

**Table 1:** Kinetic Parameters of the FRET Peptide Series Abz-XLRSSKQ-EDDnp, Abz-KXRSSKQ-EDDnp, Abz-KLRXSKQ-EDDnp, and Abz-KLRSXKQ-EDDnp Hydrolysis by Recombinant KLK2 to Characterize the Preferences of S_3_, S_2_, S′1, and S′_2_ Subsites, Respectively^a^

	Abz-XLRSSKQ-EDDnp			Abz-KXRSSKQ-EDDnp		
X	*k*_cat_ (s^–1^)	*K*_m_ (μM)	*k*_cat_/*K*_m_ (s^–1^ mM^–1^)	*k*_cat_ (s^–1^)	*K*_m_ (μM)	*k*_cat_/*K*_m_ (s^–1^ mM^–1^)
G				1.0	12.4	81
A				2.2	4.8	458
V	17.9	4.7	3808	9.9	8.5	1165
L				11.3	6.5	1738
I				4.7	4.5	1044
S	**29.8**	**3.0**	**9933**	1.1	9.1	121
T				3.6	10.5	343
N	26.7	7.8	3423	3.3	10.1	327
Q	25.7	5.9	4356	6.4	8.3	771
E	7.4	7.6	974	1.0	9.1	110
R	22.3	3.9	5718	8.7	5.2	1673
K	11.3	6.5	1738	3.0	7.2	417
H	17.2	4.2	4095	4.7	9.6	490
F	9.6	6.6	1455	4.1	1.6	2563
Y				**12.8**	**5.6**	**2304**

	Abz-KLRXSKQ-EDDnp			Abz-KLRSXKQ-EDDnp		
X	*k*_cat_ (s^–1^)	*K*_m_ (μM)	*k*_cat_/*K*_m_ (s^–1^ mM^–1^)	*k*_cat_ (s^–1^)	*K*_m_ (μM)	*k*_cat_/*K*_m_ (s^–1^ mM^–1^)
G	19.9	7.3	2120	3.5	4.5	778
A				12.2	3.8	3211
V	8.1	2.2	3682	15.3	4.2	3643
L	6.7	3.3	2030	8.5	2.8	3036
I	**7.2**	**1.8**	**4000**	4.4	2.8	1571
S	11.3	6.5	1738	11.3	6.5	1738
N	14.8	8.6	1733	4.9	8.8	557
Q	8.5	6.1	1393	6.5	5.2	1250
E	10.3	6.6	1561	7.1	8.0	888
R	8.2	5.4	1519	3.2	3.6	888
H	8.0	4.3	1860	4.4	5.9	1221
F	3.5	1.2	2917	**12.4**	**2.4**	**5167**

a The following conditions were used: pH 8.0, 20 mM Tris–HCl, 1 M sodium citrate at 37 °C, and KLK2 molar concentration = 1.6 nM. The kinetic parameters were obtained by running three enzymatic reactions, and the standard errors were 5% or less. The kinetic parameters for the best substrates are in bold and underlined.

The sequence SYRIF contains the residue of the best substrate in each series. The BLAST localized the sequence SYRIF in the following human: (a) plasma membrane calcium-transporting ATPase, (b) methyl-CpG-binding domain protein 4, (c) IFNAR2-IL10RB readthrough, and (d) ectodomain of IL-10R2. Table 2 shows the partial sequences of these proteins containing the segment SYRIF. The ectodomain of the IL-10R2 protein is the protein segment of IL-10R2 that is a common part of the heterodimeric structures constituting the ternary complexes of all class 2 cytokine (IL-10, IL-22, IL-26, IL-28, and IL-29) receptors, exposed to the extracellular environment and prone to interact with KLK2. This particular situation does not occur with the other three proteins.

**Table 2:** Results of the BLAST Program for Short Input Sequence SYRIF

protein	partial protein sequence
**plasma membrane** calcium-transporting **ATPase**	^541^VGNKTECALL GLLLDLKRDY QDVRNEIPEE ALYKVYTFNS VRKSMSTVLK NSDG^**595**^***SYRI***^**599**^***F*** S accession: NP_001353453.1
methyl-CpG-binding **domain protein 4**	^541^GND^**544**^***SYRI***^**548**^***F***CV NEWKQVHPED HKLNKYHDWL WENHEKLSLS accession: NP_003916.1
IFNAR2-IL10RB **readthrough**	^241^VPPPENVRMN SVNFKNILQW ESPAFAKGNL ***TFTAQYL***^**278**^**SYR I**^**282**^**F** QDKCMNTT accession: NP_001401434
IL-10 receptor chain-2 (IL-10R2)	^11^GCLLVSALGM VPPPENVRMN SVNFKNILQW ESPAFAKGNL TFTAQYL^***58***^***SYRI***^***62***^***F*** QDKCMNTT accession: NP_000619.3

Abz-SYRIFQ-Q-EDDnp and seven other FRET peptides derived from the IL-10R2 ectodomain containing R or K and the two preceding and following amino acids (see the legend of Table 3) were synthesized and assayed as substrates for KLK2, whose kinetic parameters are shown in Table 3. Abz-SYRIFQ-Q-EDDnp was the best substrate, followed by Abz-TLRVRAE-Q-EDDnp. The peptide Abz-LSKYGD-Q-EDDnp with lysine was resistant to hydrolysis.

**Table 3:** Kinetic Parameters of KLK2 Hydrolysis of FRET Peptides Derived from the IL-10R2 Ectodomain Containing Arginine (R) and Lysine (K)^a^

fragment	position in IL-10R2	FRET peptide	k_cat_ (s^–1^)	*K*m (μM)	k_cat_/*K*m (mM s–1)
**1**	^26^NVRM^30^N	Abz-NVR↓^b^MN-Q-EDDnp	1.28	0.94	1362
**2**	^58^SYRI^62^F	Abz-SYR↓^b^IFQ-Q-EDDnp	7.3	0.95	7684
**3**	^79^LSKYG^84^D	Abz-LSKYGD-Q-EDDnp	no hydrolysis		
**4**	^86^TLRVRA^92^E	Abz-TLR↓^b^VR↓^b^AE-Q-EDDnp	2.47	1.34	1843
**5**	^128^HMRF^132^L	Abz-HMR↓^b^FL-Q-EDDnp	0.51	0.51	1000
**6**	^177^VLRN^181^L	Abz-VLR↓^b^NL-Q-EDDnp	1.61	0.39	4
**7**	^190^QVRG^194^F	Abz-QVR↓^b^GF-Q-EDDnp	0.77	1.15	670
**8**	^195^LPDRN^200^K	Abz-LPDRNK-Q-EDDnp	no hydrolysis		

a Complete ectodomain sequence of human IL-10R2 with highlighted arginines (R), one lysine (K), and the sequence ^58^SYRI^62^F. 1 MAWSLGSWLG GCLLVSALGM VPPPENV***R***MN SVNFKNILQW ESPAFAKGNL TFTAQYL^**58**^***SYR***. 61 *I*^62^**F**QDKCMNTT LTECDFSSLS **K**YGDHTL***R***V***R*** AEFADEHSDW VNITFCPVDD TIIGPPGMQV. 121 EVLADSLHM***R*** FLAPKIENEY ETWTMKNVYN SWTYNVQYWK NGTDEKFQIT PQYDFEVL***R***N. 181 LEPWTTYCVQ V***R***GF LPD***R***NK AGEWSEPVCE QTTHDETVPS.

b ↓ Represents the cleavage sites.

### Effects of Salts, pH, and Glycosaminoglycans on KLK2 Activity

**Figure 1:**
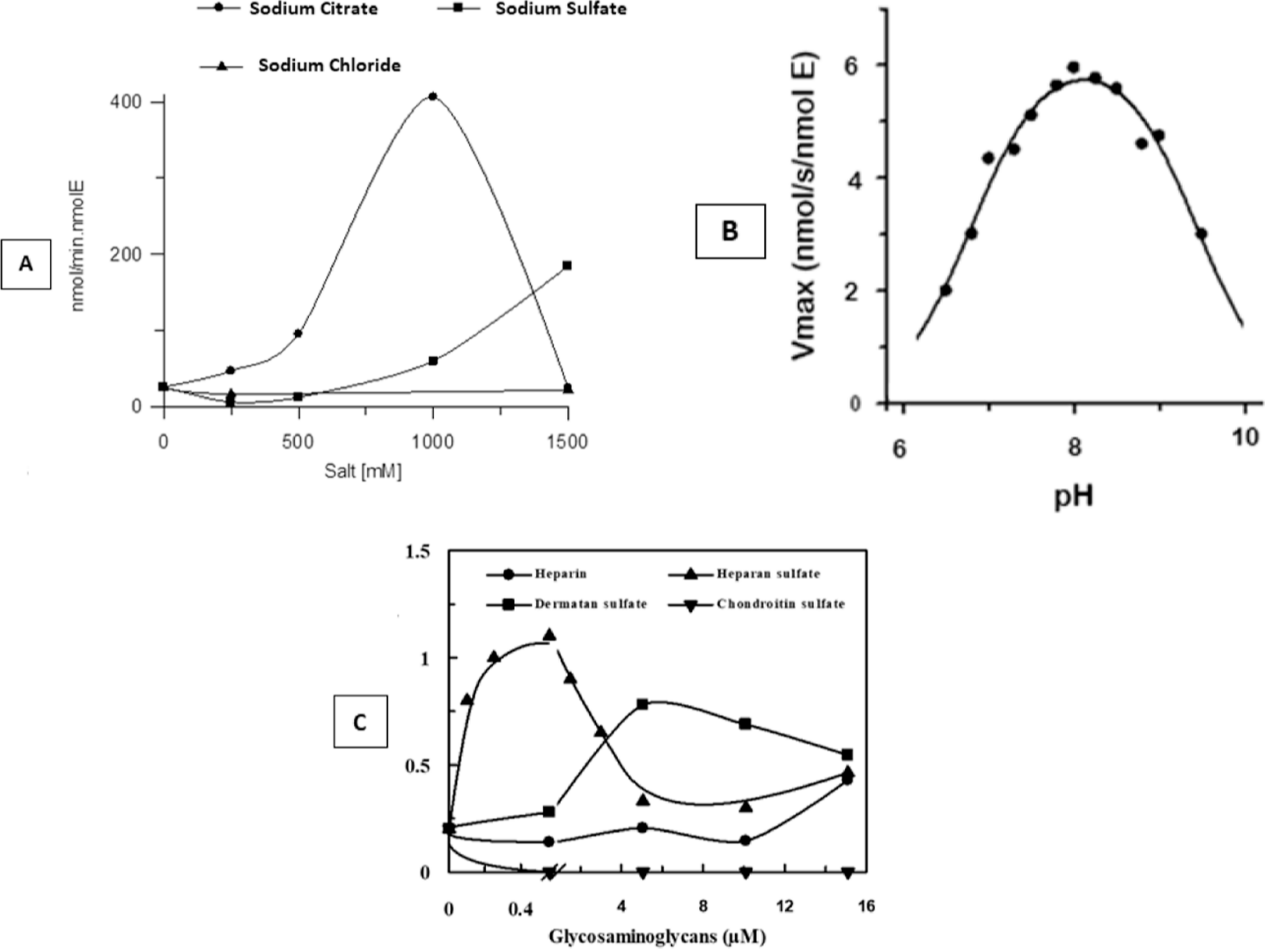
KLK2 hydrolysis of Abz-KLRSSKQ-EDDnp. (A) Effects of sodium citrate, sodium sulfate, and sodium chloride on the FRET-peptide hydrolysis by KLK2. (B) pH profile for the KLK2 activity. (C) Effects of glycosaminoglycans on KLK2 activity. Assays were carried out in 20 mM Tris–HCl, 1 mM EDTA, and 5.0 μM substrate; [E] = 1.58 nM at 37 °C and pH 8.0 for experiments in (A and C).

Figure 1A shows the effects of sodium citrate, sodium sulfate (kosmotropic salts), and sodium chloride (neutral salt) on the KLK2 activity. These results indicate that the KLK2 activity strictly depends on the order-making effect of the kosmotropic salts and its nature since sodium citrate is a ten times more efficient activator of KLK2 than sodium sulfate. The pH profile of KLK2 activity (Figure 1B) shows that KLK2’s best activity is in the pH 7.5–8.5 range. Heparan sulfate and dermatan sulfate activate KLK2 in sub- and micromolar concentrations, respectively, and heparin showed almost no effect, whereas chondroitin sulfate was inhibitory (Figure 1C).

### Effects of KLK2 on BMDM Cells

**Figure 2:**
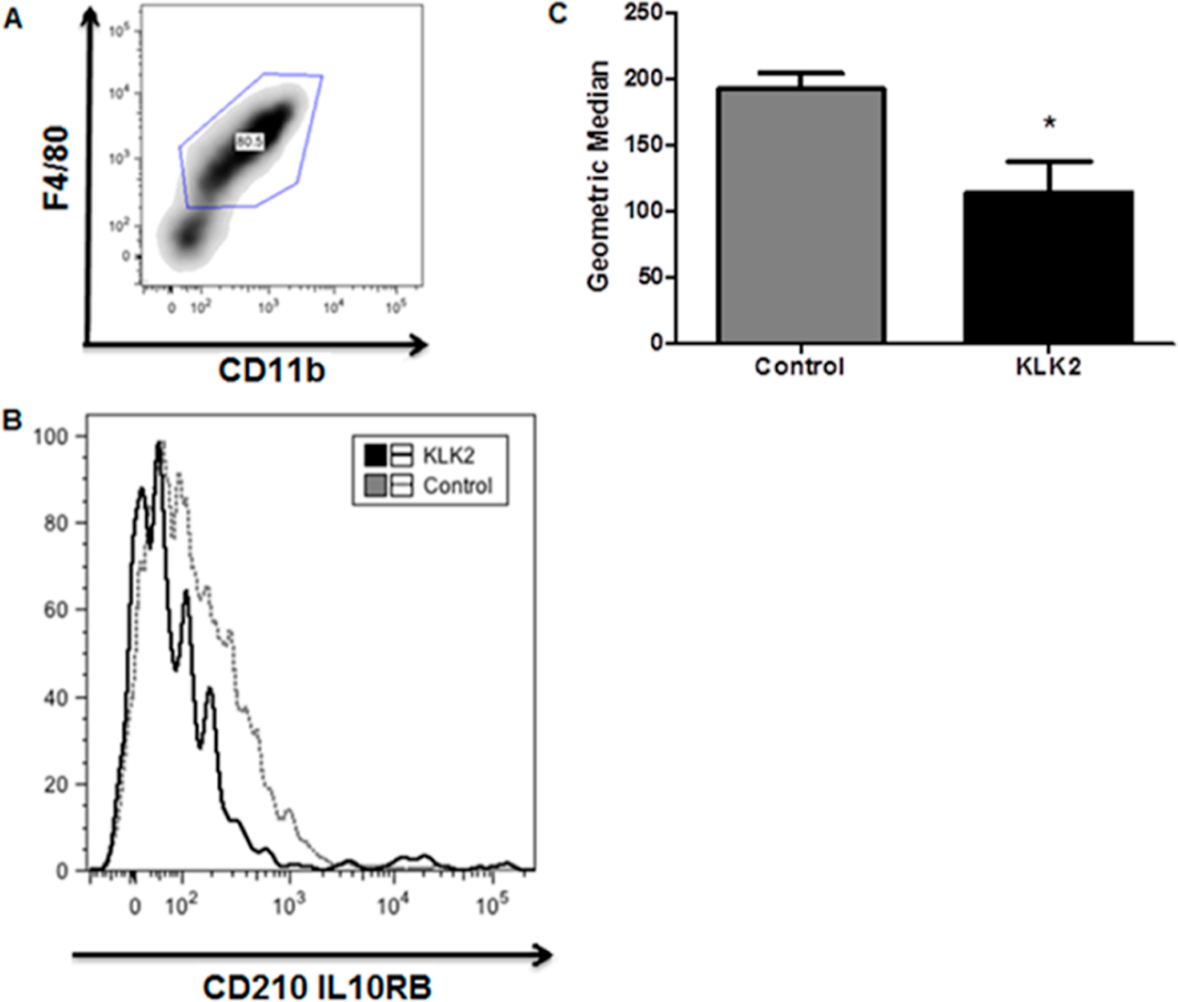
KLK2 decreases the level of IL-10RB detection in BMDM. BMDM cells were treated with KLK2 (7.5 μg/mL) for 2 h, and the cell surface markers F4/80^+^ (PercP-Cys, BD Bioscience) and CD11b^+^ (APC, BD Bioscience) were used to select the cells, and IL-10RB (Alexa Fluor 488, Abcam) expression was evaluated on these macrophages. (B) The fluorescence intensity of IL-10RB expression was evaluated on F4/80^+^ and CD11b^+^ macrophages, as determined by FACS and represented in the histogram. Control and KLK2 groups are shown. (C) The geometric mean of IL-10RB fluorescence intensity in control and KLK2 groups was extracted from FlowJo Software (Tree Star, CA, USA). Bars represent the means ± SD from 3 independent experiments. *p value < 0.05 analyzed by Student’s *t*-test. See ref (18) for details and use of this analysis.

Compared to the control group, BMDM cells treated with KLK2 showed a significant reduction of IL-10R2 on the surface, as detected by flow cytometry (FACS analysis) (Figure 2), indicating that KLK2 acts directly on IL-10R2.

**Figure 3:**
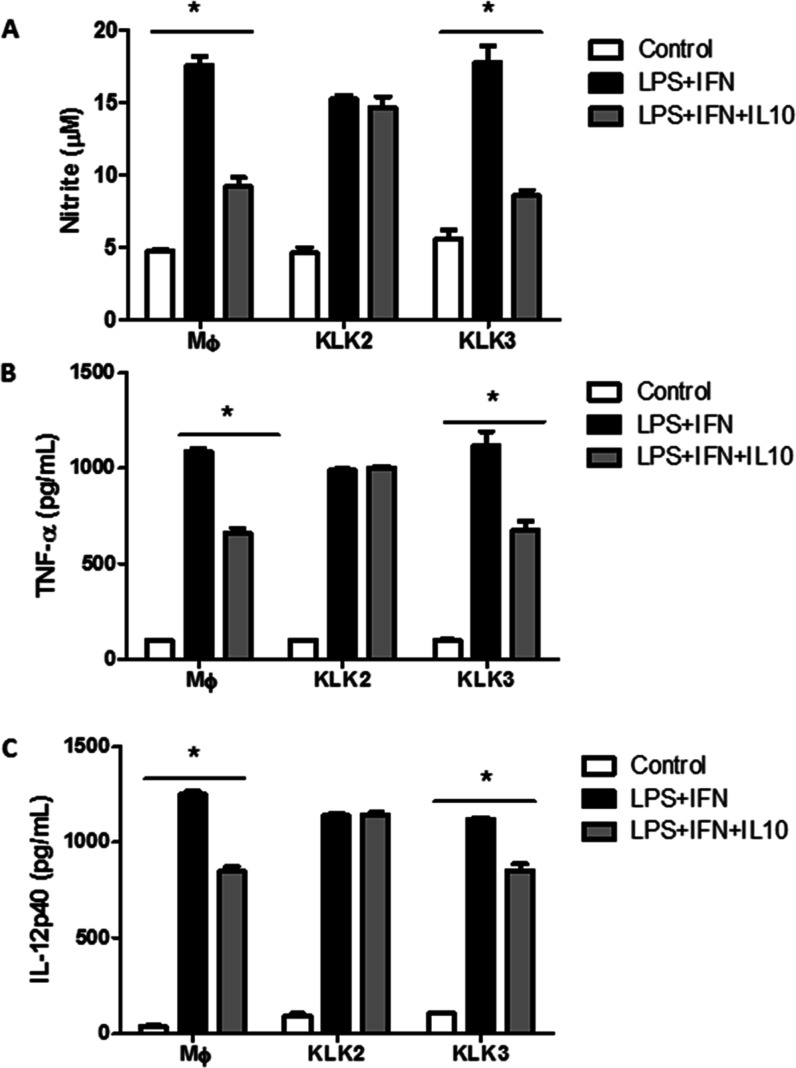
KLK2 converts BMDM cells to a pro-inflammatory status characterized by maintaining NO, TNF-α, and IL-12p40 levels, even in the presence of recombinant IL-10. BMDM cells from C57Bl/6 mice were incubated in a 96-well plate (1 × 10^5^ cells) with KLK2 (7.5 μg/mL) or KLK3 (7.5 μg/mL) for 2 h. After this, cells were washed and incubated with or without lipopolysaccharide (LPS) (200 ng/mL), IFN-γ (IFN), and recombinant IL-10 (IL10). (A) NO quantified in the culture supernatant by the Griess method. (B) TNF-α and (C) IL-12 p40 cytokines were quantified by ELISA in culture supernatants. MΦ = control macrophages. * *p*-value <0.001, analyzed by ANOVA with Tukey’s multiple comparisons.

This effect was further confirmed in a BMDM activation assay with LPS and IFN-γ. In this system, recombinant IL-10 reduced the generation of NO, TNF-α, and IL-12 p40, but in BMDM pretreated with KLK2, these IL-10 inhibitory effects were not observed (Figure 3A–C). Similar experiments with KLK3 did not show these effects, suggesting resistance of the extracellular domain of IL-10R2 to hydrolysis by KLK3. This result was confirmed by the resistance to hydrolysis by KLK3 of the IL-10R2-derived peptides containing the F or Y amino acids, whose sequences (Table 1S) were synthesized and tested with KLK3. These data indicate that only KLK2 decreases the cell surface expression of IL-10R2 on macrophages, thus contributing to a pro-inflammatory response.

## Discussion

**Figure 4:**
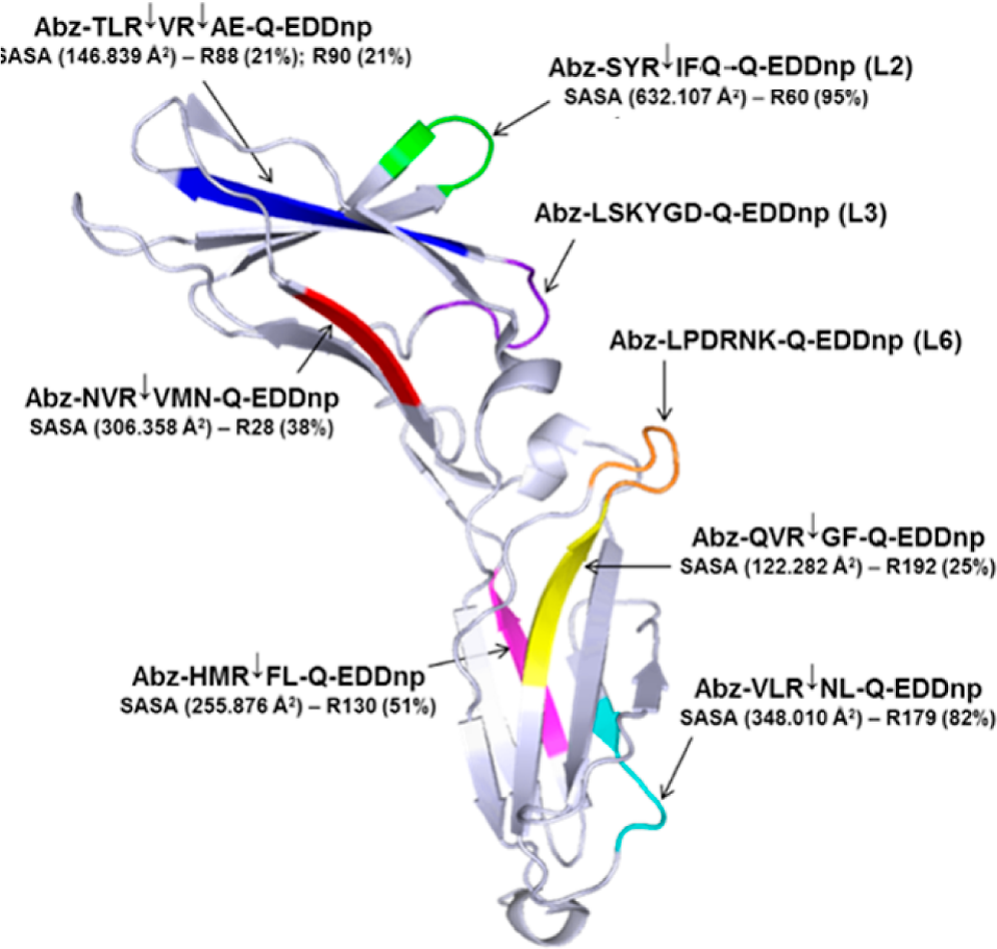
Molecular surface structure of IL-10R2 (PDB/ID 3LQM: A) ectodomain with highlighted loops. The structure of the IL-10R2 (PDB id: 3LQM) ectodomain with highlighted segments corresponding to the synthetic peptides containing R and K (loop 3). The solvent-accessible surface area (SASA) of the L2 loop corresponding to the synthetic peptide Abz-SYRIFQ-Q-EDDnp and cleaved efficiently by KLK2 (*k*_cat_/*K*_m_: 7684 mM s^–1^) has a high SASA of 692 Å2 and ^60^R with 95% of exposition to solvent. SASA calculation and the structure visualization were performed in PyMol (The PyMOL Molecular Graphics System Version 2.0, Schrödinger, LLC).

The efficient hydrolysis by KLK2 of peptides Abz-SYRIFQ-Q-EDDnp and Abz-TLRVRAE-Q-EDDnp that have the amino acid sequences present in the ectodomain of IL-10R2 led us to hypothesize that this chain of the IL-10 receptor could be susceptible to hydrolysis by KLK2. The sequence ^58^SYRIF^62^ forms loop L2 in IL-10R2 exposed to solvent, as observed by the crystal structure of IL-10R2,^20,21^ as shown in Figure 4. The IL-10R2 structure contains other loops that connect their structural β strands and, like loop L2, are involved in IL-10 binding. The sequence ^86^TLRVRAE^92^ forms a β-strand structure close to loop 2, and the corresponding FRET peptide Abz-TLRVRAE-Q-EDDnp is also well hydrolyzed by KLK2. ^195^LPDRN^200^K sequence is part of loop L6 (Figure 4), but the peptide Abz-LPDRNK-Q-EDDnp was resistant to KLK2, possibly due to the unfavorable effect of the negatively charged aspartyl residue at the P_2_ position.

These structural details and the observed effects of KLK2 decreasing the detection of IL-10R2 on the surface of BMDM cells (Figure 2), thus favoring their pro-inflammatory status (Figure 3), strongly suggest that the IL-10R2 ectodomain is a substrate for KLK2. The hydrolysis of IL-10R2 possibly occurs in a cascade beginning at loop 2, which is very exposed and has a very susceptible sequence for the hydrolytic activity of KLK2. These effects of KLK2 on IL-10R2 impair the anti-inflammatory activity of IL-10, and in the prostate, where KLK2 is almost exclusively expressed, the suppression of IL-10 activity can induce prostate inflammation, hyperplasia, and cancer.^22−24^ This possibility is supported by the observed correlation in the Korean population of IL-10R2 single nucleotide polymorphisms with benign prostate hyperplasia^25^ and also in chronic prostatitis and pelvic pain syndrome in men, which were associated with a genotype with low IL-10 production.^26,27^ It is relevant to point out that IL-10R2 is a common part of the heterodimeric structures constituting the ternary complexes of all class 2 cytokine (IL-10, IL-22, IL-26, IL-28, and IL-29) receptors. This means that the hydrolysis of IL-10R2 by KLK2 can interfere with the functions of all class 2 cytokines, at least in the prostate. It is noteworthy that the peculiar prostate extracellular environment, particularly in inflammatory conditions, namely, the elevated sodium citrate concentration,^26,28^ pH around 8 of prostatic fluid,^12,13^ and the presence of glycosaminoglycans, all lead KLK2 to its best hydrolytic activity.

## Conclusions

In summary, we reported studies on the KLK2 substrate specificity and present data, indicating that the extracellular IL-10R2 component is a KLK2 substrate. The consequent inactivation of IL-10R2 by KLK2 was confirmed by the reduction of IL-10 activity in BMDM cells pretreated with KLK2. Notably, the activation of KLK2 by extracellular components of the prostate environment, such as sodium citrate, heparan sulfate, and dermatan sulfate, controls the quantities and structures of the prostate cells. It is relevant to point out that at pH 8, KLK2 has its full activity, but this is the pH of prostate secretion under inflammatory conditions. It contrasts with the acidic pH in a normal prostate, where KLK2 is less active. Therefore, KLK2 may have a very significant role in inflammation and hyperplasia as well as prostate tumorigenesis, and KLK2 inhibitors could be promising drugs in these prostate dysfunctions.

## Supporting Information Available

FRET peptides; HPLC chromatograms and mass spectra of the peptides; and cleavage products of reactions of IL10-R2 with KLK2 (PDF)

## Accession Codes

Human prostate kallikrein-2 (KLK2): **P 20151**. Human prostate kallikrein-3 (KLK3): **P 07288**. IL-10 receptor2 (IL-10R2): **Q 08334**TNF-α: **P 01375**. IL-12 p40: **P 29460**. Plasma membrane calcium-transporting ATPase1: **P20020**. Methyl-CpG-binding domain protein 4: **O95243** · **MBD4_HUMAN**.
