# Supplementary material for: Extracellular Domain of IL-10 Receptor Chain-2 (IL-10R2) and Its Arginine-Containing Peptides Are Susceptible Substrates for Human Prostate Kallikrein-2 (KLK2)
Source: Biochemistry. 2024 Aug 6;63(16):2023–9. doi: 10.1021/acs.biochem.4c00292 (PMC11339918; doi:10.1021/acs.biochem.4c00292)

The extracellular domain of IL-10 receptor chain-2 (IL-10R2) and its arginine-containing peptides are susceptible substrates for human prostate kallikrein-2 (KLK2).

Juliana R. Oliveira[a], José Thalles Lacerda[a], Tarciso A. Sellani[c], Elaine G Rodrigues[c], Luiz R Travassos[c], Maria A Juliano[a] and Luiz Juliano[a,b]*.

[a]Department of Biophysics, Escola Paulista de Medicina, Federal University of São Paulo, Rua Três de Maio 100, 04044-20 - São Paulo, Brazil

[b]International Research Center, A.C. Camargo Cancer Center, Rua Taguá, 440, 01509-010 São Paulo, Brazil

[c]Department of Microbiology, Immunology and Parasitology, Escola Paulista de Medicina, Federal University of São Paulo, Rua Botucatu 862, 04023-901 - São Paulo, Brazil.

Supporting information

Table 1S.

FRET peptides derived from IL-10R2 ectodomain containing F or Y were resistant to hydrolysis by KLK3

FRET Peptides with **F**	FRET Peptides with **Y**
Abz-MNSVN**F**KNIL-Q-EDDnp	Abz-FTAQ**Y**LS**Y**R-Q-EDDnp
Abz-WESPA**F**AKGN-Q-EDDnp	Abz – AQYLS**Y**RIF-Q-EDDnp
Abz-KGNLT**F**TAQY-Q-EDDnp	Abz-SSLSK**Y**GDHT-Q-EDDnp
Abz-LSYRI**F**QDKA-Q-EDDnp	Abz-KIENE**Y**ETWT-Q-EDDnp
Abz-LTEAD**F**SSLS-Q-EDDnp	Abz-TMKNV**Y**NSWT-Q-EDDnp
Abz-RVRAE**F**ADEH-Q-EDDnp	Abz-NSWT**Y**NVQ-Q-EDDnp
Abz-HSDWVNIT**F**APVD-Q-EDDnp	Abz-NVQ**Y**WKNG-Q-EDDnp
Abz-SLHMR**F**LAPK-Q-EDDnp	Abz-QITPQ**Y**DFEV-Q-EDDnp
Abz-GTDEK**F**QITP-Q-EDDnp	Abz – EPWTT**Y**AVQR-Q-EDDnp
Abz-TPQYD**F**EVLR-Q-EDDnp	
Abz-VQVRG**F**LPDR-Q-EDDnp	

Conditions of reactions: 2 nM KLK3, 20 mM Tris-HCl, 1 mM EDTA, 1.5 M sodium citrate, pH 7.8, at 37 ^{o}C.

The sequence of FRET peptide with F or Y in the middle of the sequence can be identified in the IL-10R2 ectodomain sequence around **F** or **Y**, as shown below. Cysteines were substituted by Alanine (A)

Positions of **F**

1 MAWSLGSWLG GCLLVSALGM VPPPENVRMN SVN**F**KNILQW ESPA**F**AKGNL T**F**TAQYLSYR.

61 I**F**QDKCMNTT LTECD**F**SSLS KYGDHTLRVR AE**F**ADEHSDW VNIT**F**CPVDD TIIGPPGMQV.

121 EVLADSLHMR **F**LAPKIENEY ETWTMKNVYN SWTYNVQYWK NGTDEK**F**QIT PQYDFEVLRN.

181 LEPWTTYCVQ VRG**F** LPDRNK AGEWSEPVCE QTTHDETVPS.

Positions of **Y**

1 MAWSLGSWLG GCLLVSALGM VPPPENVRMN SVNFKNILQW ESPAFAKGNL TFTAQ**Y**LS**Y**R.

61 IFQDKCMNTT LTECDFSSLS K**Y**GDHTLRVR AEFADEHSDW VNITFCPVDD TIIGPPGMQV.

121 EVLADSLHMR FLAPKIENE**Y** ETWTMKNV**Y**N SWT**Y**NVQYWK NGTDEKFQIT PQ**Y**DFEVLRN.

181 LEPWTT**Y**CVQ VRGF LPDRNK AGEWSEPVCE QTTHDETVPS.

FRET peptide synthesis and purification

An automated bench-top simultaneous multiple solid-phase peptide synthesizer (PSSM 8 system from Shimadzu) was used to synthesize all the peptides by the Fmoc procedure, described in detail in reference (Korkmaz B et al., Nat Protoc. 2008;3:991-1000. DOI: 10.1038/nprot.2008.63)

The final peptides were removed from the resin and deprotected by TFA, followed by lyophilization. Then, they were dissolved in trifluoroacetic acid (TFA)/H2O (1:1000) and purified by semipreparative HPLC using an Econosil C-18 column (10 µ, 22.5 x 250 mm) and a two-solvent system: (A) trifluoroacetic acid (TFA)/H2O (1:1000) and (B) TFA/acetonitrile (ACN)/H2O (1:900:100). The column was eluted at a flow rate of 5 ml/min with a 10 (or 30) - 50 (or 60)% gradient of solvent B over 30 or 45 min, or at a flow rate of 8 ml/min with a 0 (or 5) – 90% gradient of solvent B over 70 min.

The Analytical HPLC was performed using a binary HPLC system from Shimadzu with a SPD-10AV Shimadzu uv-vis detector, coupled to an Ultrasphere C-18 column (5 µ, 4.6 x 150 mm) which was eluted with solvent systems A1 (H3PO4/H2O, 1:1000) and B1 (ACN/ H2O/H3PO4, 900:100:1) at a flow rate of 1.0 ml/min and a 5%-80% gradient of B1 over 12 min. The HPLC column eluates were monitored by their absorbance at 220 nm. The molecular weight and purity of synthesized peptides were checked by mass spectrometry LC-MS-2020 Single Quadrupole (Shimadzu). All peptides must have >95% purity by this HPLC analysis or then be repurified.

FRET peptides derived from IL-10R2 ectodomain containing arginine (R) and cleaved by KLK2

1. HPLC chromatograms and mass spectra of the peptides

Abz-NVRMNQ-EDDnp

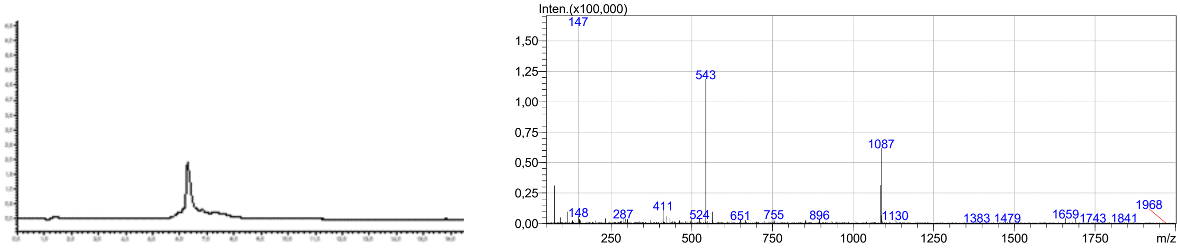

Abz-SYRIFQQ-EDDnp

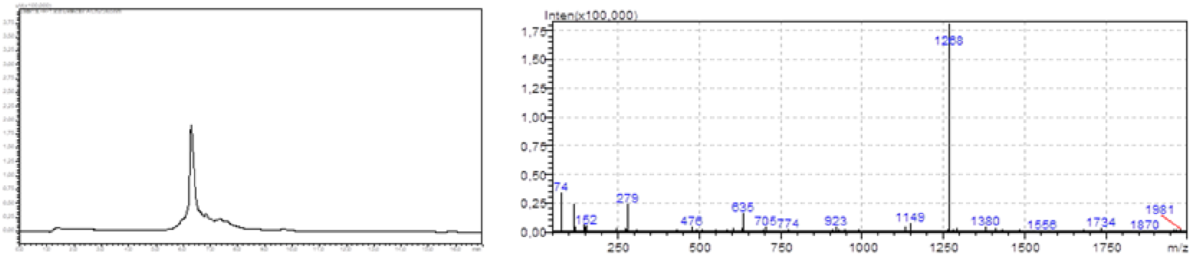

Abz-TLRAEQ-EDDnp

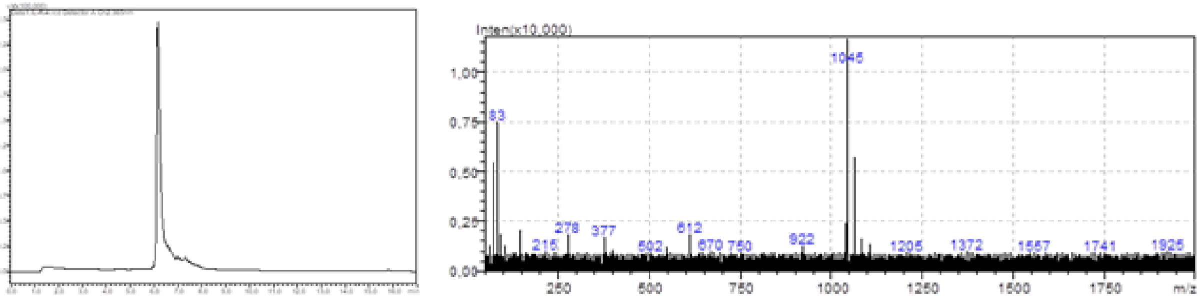

Abz-HMRFLQ-EDDnp

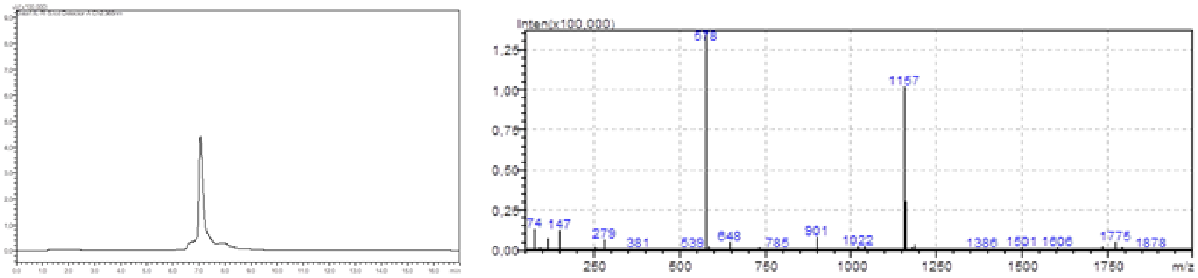

Abz-VLRNLQ-EDDnp

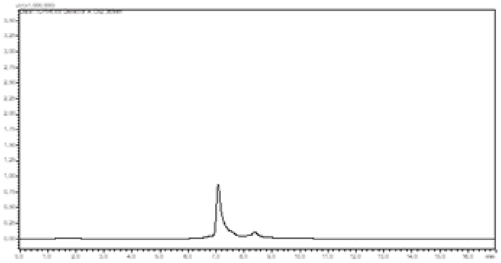

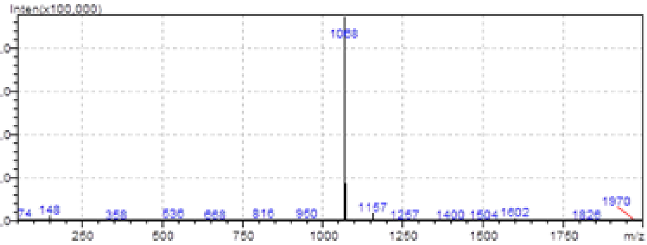

Abz-QVRGFQ-EDDnp

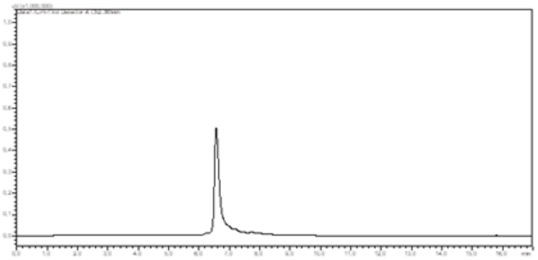

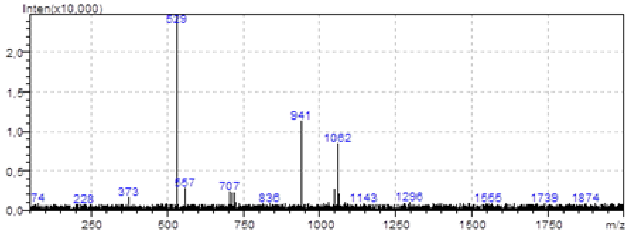

Abz-LPDRNKQ-EDDnp

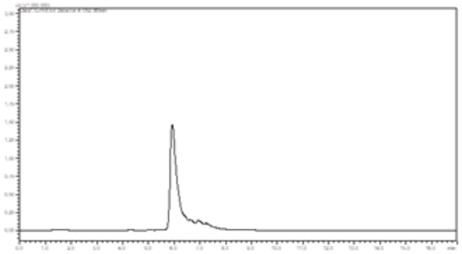

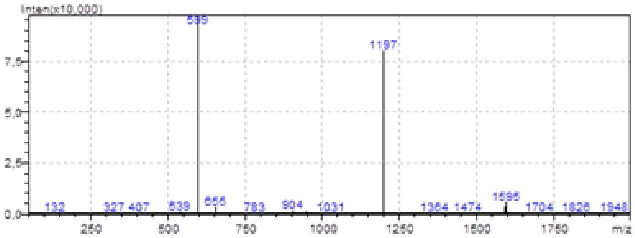

2. Cleavage products of reactions of IL10-R2 with KLK2: HPLC chromatograms and mass spectra

Abz-NVR↓MNQ-EDDnp

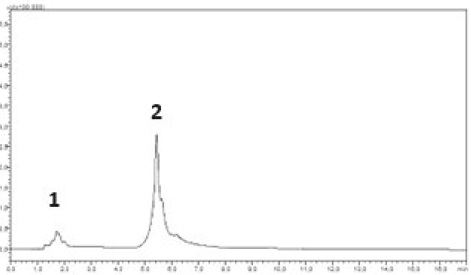

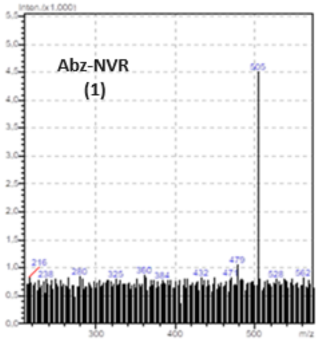

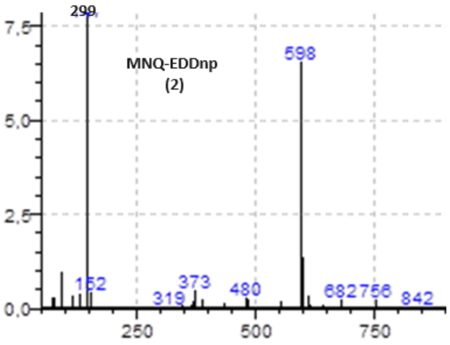

Abz-SYR↓IFQQ-EDDnp

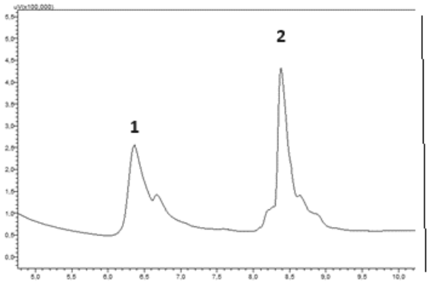

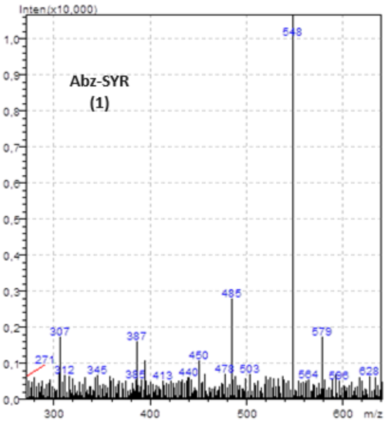

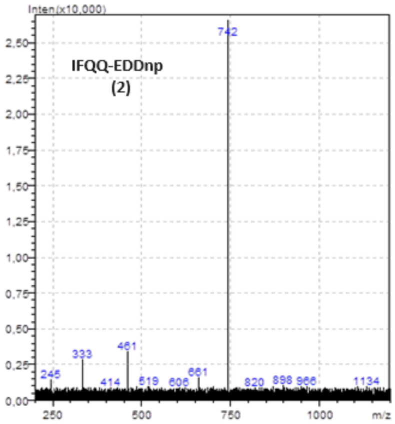

Abz-TLR↓AEQ-EDDnp

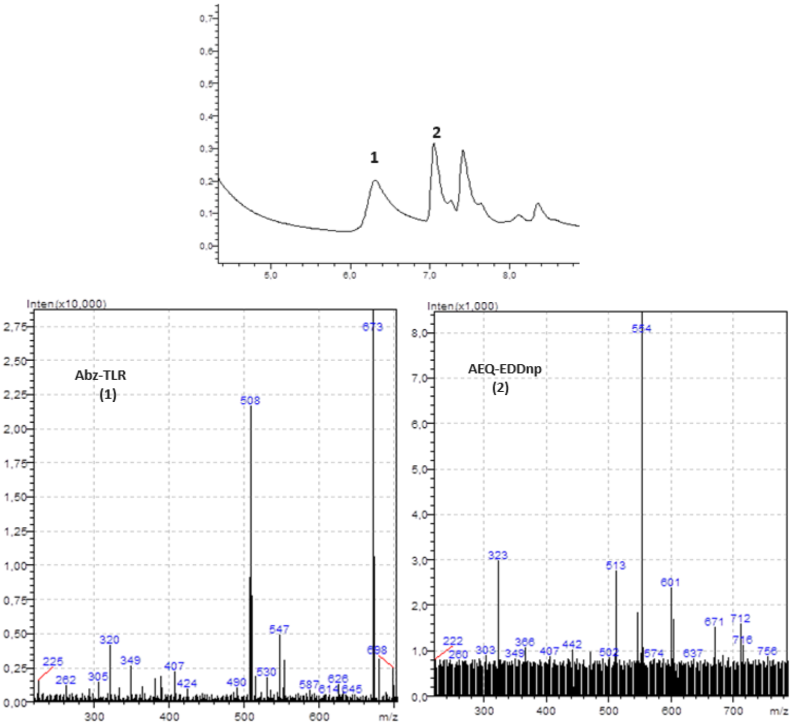

Abz-HMR↓FLQ-EDDnp

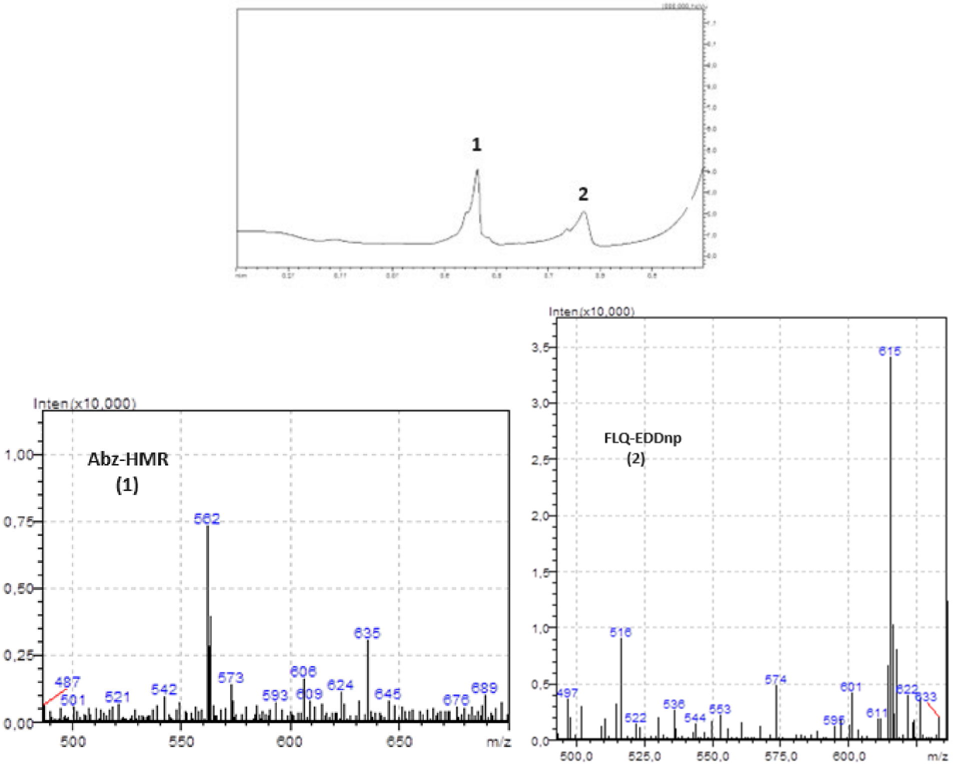

Abz-VLR↓NLQ-EDDnp

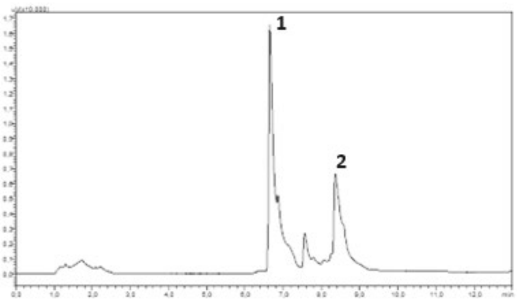

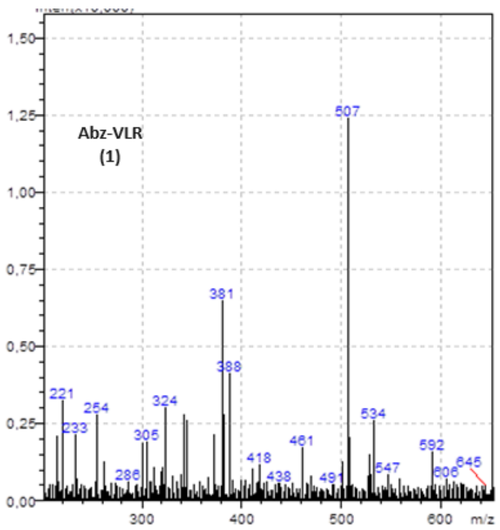

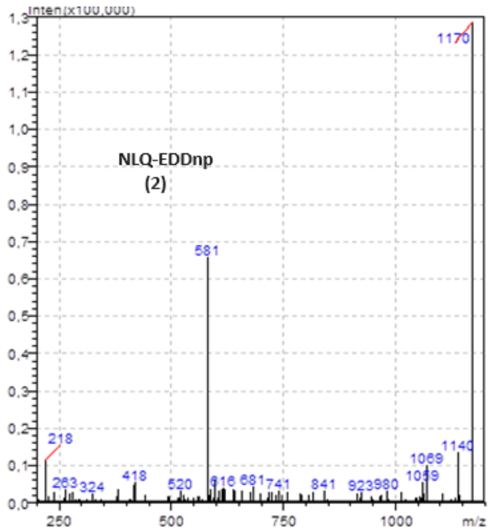

Abz-QVR↓GFQ-EDDnp

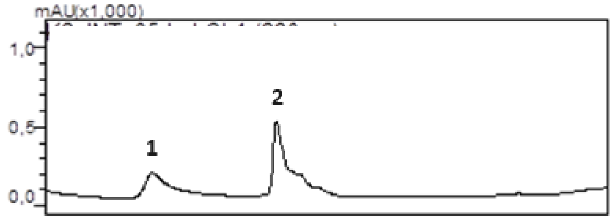

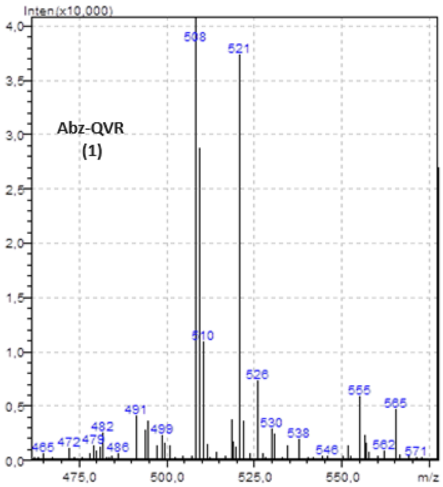

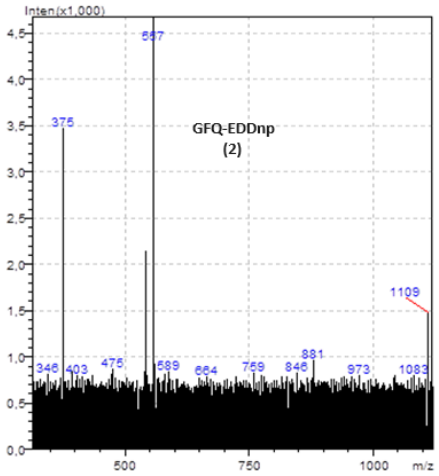

Supplement: Supplementary file 1 — bi4c00292_si_001.pdf [file bi4c00292_si_001.pdf]
